# Liquid biopsy: paving a new avenue for cancer research

**DOI:** 10.1080/19336918.2024.2395807

**Published:** 2024-09-01

**Authors:** Keerthi Kurma, Zahra Eslami-S, Catherine Alix-Panabières, Laure Cayrefourcq

**Affiliations:** a Laboratory of Rare Human Circulating Cells (LCCRH), University Medical Centre of Montpellier, Montpellier, France; b CREEC/CANECEV, MIVEGEC (CREES), University of Montpellier, CNRS, IRD, Montpellier, France; c European Liquid Biopsy Society (ELBS), Hamburg, Germany

**Keywords:** Liquid biopsy, biomarkers, circulating tumor cells, circulating free or tumor DNA, circulating cell-free RNA, extracellular vesicles, tumor-educated platelets

## Abstract

The current constraints associated with cancer diagnosis and molecular profiling, which rely on invasive tissue biopsies or clinical imaging, have spurred the emergence of the liquid biopsy field. Liquid biopsy involves the extraction of circulating tumor cells (CTCs), circulating free or circulating tumor DNA (cfDNA or ctDNA), circulating cell-free RNA (cfRNA), extracellular vesicles (EVs), and tumor-educated platelets (TEPs) from bodily fluid samples. Subsequently, these components undergo molecular characterization to identify biomarkers that are critical for early cancer detection, prognosis, therapeutic assessment, and post-treatment monitoring. These innovative biosources exhibit characteristics analogous to those of the primary tumor from which they originate or interact. This review comprehensively explores the diverse technologies and methodologies employed for processing these biosources, along with their principal clinical applications.

## Introduction

Cancer is a pressing global health concern. It is among the most prevalent causes of death, and its prevalence is increasing worldwide [1] (World Health Organization. Cancer https://www.who.int/news-room/fact-sheets/detail/cancer. Accessed Nov 2021). The impact of cancer extends to the quality of life, imposing significant physical, emotional, and financial burdens on patients, their families, and the healthcare system. Despite extensive research aimed at elucidating the pathophysiology of cancer and the notable advancements in cancer management, the majority of metastatic cancers remain incurable, with 5-year survival rates falling below 25% [2] (World Health Organization. Cancer. https://www.who.int/news-room/fact-sheets/detail/cancer. Accessed Apr 2022). Furthermore, cancer is often diagnosed at advanced stages when treatment tends to have limited efficacy. As cancer progresses, tumors tend to diversify, significantly influencing their response to therapy. Although treatment can eliminate dominant, susceptible cancer cells, a small subset of resilient cells may persist and continue to proliferate. Over the past decade, precision medicine has emerged as a ground breaking approach in oncology research. This approach revolves around sequencing each patient’s tumor and provides tailored insights [3]. The conventional benchmark for diagnosing tumors and conducting molecular profiling is tissue biopsy. However, this approach is burdened with a range of challenges, including invasiveness and its associated issues such as bleeding, injury, infection, pain, limited access to tissue, and concerns related to sample adequacy, involving insufficient volume and suboptimal sample quality [4,5].

Furthermore, cancer diagnosis primarily relies on tissue biopsies, which only capture a partial representation of the entire tumor mass and often fail to fully encapsulate the intricacies of the disease [6]. Moreover, in the case of metastasis, where tumors have disseminated and continuously adapted both spatially and temporally in response to treatment, the need for multiple biopsies becomes evident to gain a comprehensive understanding of the tumor’s behavior [7]. Similarly, the ongoing acquisition of biopsy samples through invasive procedures during treatment, aimed at monitoring tumor response and relapse, presents a significant challenge for tumor profiling. These limitations are overcome by liquid biopsy, which was introduced as a new diagnostic concept and offers several clinical applications [8,9].

**Figure 1.**
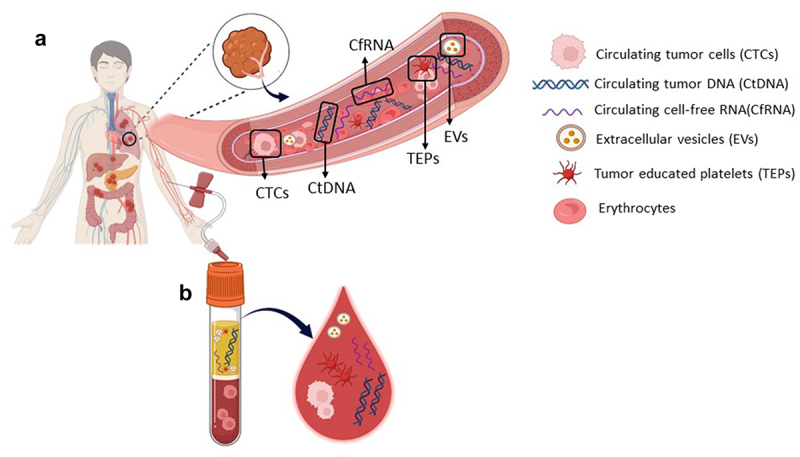
Liquid biopsy biomarkers.(a) A subset of circulating biomarkers, such as CTCs, CtDNA, CfRNA, TEPs, and EVs, enters the bloodstream from the primary tumor and/or metastatic lesions. (b) Schematic view of liquid biopsy. Blood collected from cancer patients contains circulating biomarkers. These biomarkers can provide real-time information on tumor progression, prognosis, and treatment response.

The term *‘liquid biopsy’* was coined by Alix-Panabières and Pantel in 2010 [10,11] to describe circulating tumor cells (CTCs). Subsequently, this terminology has been expanded to encompass other circulating biomarkers, such as circulating cell-free tumor DNA (ctDNA), circulating cell-free RNA (cfRNA), extracellular vesicles (EVs), and tumor-educated platelets (TEPs) [4,11–16] (Figure 1). Liquid biopsy provides enhanced sensitivity in diagnosis and ease of repeated sampling throughout treatment in a much more convenient and noninvasive way [17]. The utility of liquid biopsy extends beyond merely mirroring tissue biopsies. It is a potential tool capable of revealing distinctive and crucial insights about a patient’s cancer that traditional tissue testing cannot provide. Additionally, research has focused on the application of liquid biopsy for the early identification of tumors [18]. Taken together, these tumor-derived components can provide crucial longitudinal information and data, aiding pathologists in achieving a more precise diagnosis of both primary and metastatic tumors. Currently, more than 10,000 publications are listed under the key phrase ‘liquid biopsies’ in PubMed, targeting almost all types of cancers.

Hence, this chapter provides an overview of liquid biopsy’s involving the isolation of CTCs, ctDNA, cfRNA, EVs, and TEPs from the blood of individuals with diverse cancer types. In addition, it highlights the applications of these isolated entities in the identification, prognosis, and treatment of numerous cancer types.

## Liquid biopsy biomarkers and their current isolation technologies/methodologies

### Circulating tumor cells

The process of cancer cell dissemination throughout the body and the subsequent metastatic cascade commences with the active release of the most aggressive tumor cells into the bloodstream and/or lymphatic vessels, commonly referred to as CTCs [10,19,20]. Notably, during metastasis, cancer cells undergo epithelial-to-mesenchymal transition (EMT), a complex phenomenon marked by the disruption of intercellular connections, penetration of the basal membrane and adjacent tissues, entry into venous or lymphatic vessels leading to the formation of CTCs, persistence in the peripheral system, and subsequent processes of extravasation and proliferation at secondary sites [21,22]. EMT supports the migration of epithelial tumor cells and is believed to play a crucial role in facilitating metastasis [23]. Furthermore, in normal tissues, the loss of adhesion to the extracellular matrix triggers cell death in anchorage-dependent cells, a process known as ‘anoikis’ [24,25]. In contrast, tumor cells/CTCs that have developed resistance to anoikis can survive detachment from their primary site and navigate through the circulatory and lymphatic systems to reach distant locations [26,27]. Once in circulation, CTCs encounter various stresses inherent to this new environment, particularly immune system stress. A significant body of research has been dedicated to understanding the immunosuppressive mechanisms that enable CTCs to evade immune system surveillance [28,29]. Additionally, cancer cells can form associations with other cells, creating microemboli that aid their survival in the bloodstream. Various mechanisms of cell association have been described, including CTC clusters, which exhibit higher metastatic potential by enhancing cell survival and reducing apoptosis [30–32]; partnering with reactive platelets, which serve as a shield or camouflage against attacks by the immune system [33–35]; and clustering with white blood cells, primarily neutrophils, which might promote cell cycle progression, leading to more efficient metastasis formation [32]. The enumeration and analysis of CTCs provide valuable insights into the molecular profile of tumors and can facilitate the identification of cells that initiate metastasis [25]. A direct correlation exists between the number of CTCs in the blood, expression of specific biomarkers (e.g., cancer stem cell markers), and development of distant metastases [36,37]. CTCs serve as an optimal source for characterizing and monitoring solid cancers because they can be subjected to genome, proteome, transcriptome, and secretome analyses. Although CTCs are present in low numbers in the bloodstream, recent advancements in high-tech methods have enabled the detection and characterization of individual CTCs [12,38].

#### Enrichment and detection technologies of CTCs from liquid biopsies

**Figure 2.**
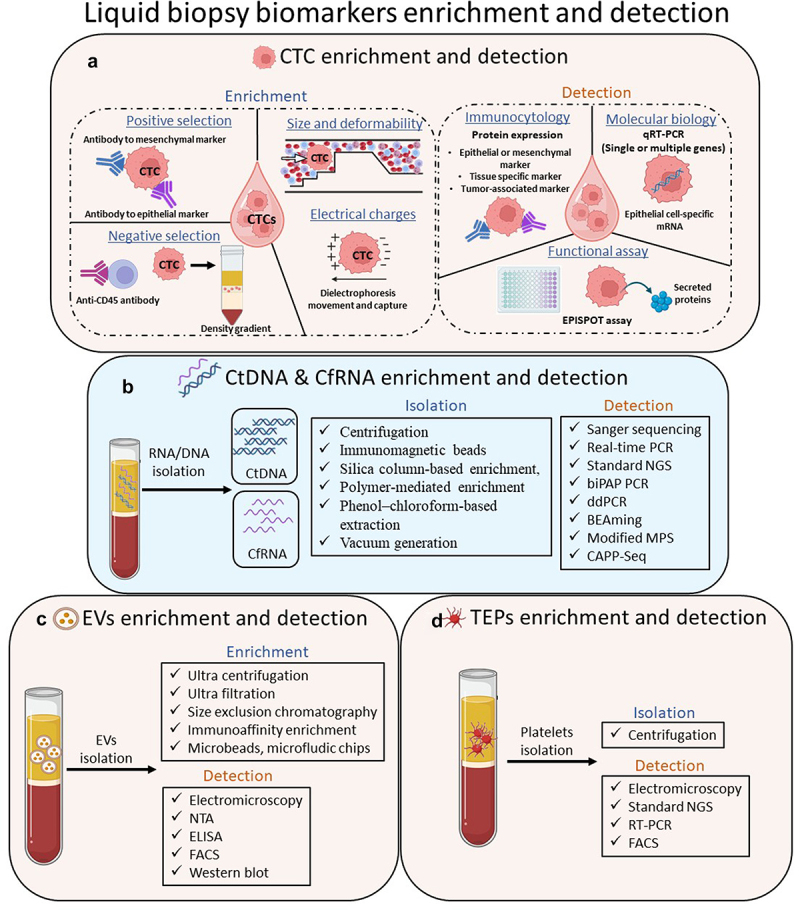
Overview of enrichment and detection methods for circulating tumor cells (CTCs), circulating tumor DNA (ctDNA), circulating cell-free tumor RNA (cfRNA), extracellular vesicles (EVs) and tumor educated platelets (TEPs).(a) CTCs in blood can be enriched using marker-dependent techniques: CTCs can be positively selected using antibodies against epithelial and/or mesenchymal proteins (such as the epithelial cell adhesion molecule (EPCAM) and/or cytokeratin) or negatively selected through depletion of leukocytes using anti-CD45 antibodies. Positive enrichment of CTCs can also be performed using assays based on CTC characteristics including size, deformability, density, and electrical charge. Following enrichment, isolated CTCs can be identified using immunocytological, molecular, or functional assays. Using immunocytological platforms, CTCs are identified by membrane and/or intracytoplasmic staining with antibodies to epithelial, mesenchymal, tissue-specific, or tumor-associated markers. Molecular technologies enable the identification of CTCs using RNA-based assays such as quantitative reverse transcription PCR (qRT-PCR), RNA sequencing, or in situ RNA hybridization. Functional assays, such as the fluoro-epithelial ImmunoSPOT (EPISPOT) assay for certain proteins secreted or shed by CTCs, can be used to detect viable CTCs based on their biological activities. (b) Tumor-associated genetic aberrations can be detected in ctDNA and cfRNA extracted from the plasma of patients with cancer. Following enrichment, the isolated ctDNA/cfRNA was detected by Sanger sequencing, real-time polymerase chain reaction (PCR), standard next-generation sequencing (NGS), bidirectional pyrophosphorolysis-activated polymerization (biPAP) PCR, droplet digital PCR (ddPCR), bead-emulsion-amplification-magnetics (BEAming), modified massively parallel sequencing (MPS), and cancer personalized profiling by deep sequencing (CAPP-Seq). (c) EVs in the blood can be enriched from the plasma of cancer patients by ultra-centrifugation, ultrafiltration, size exclusion chromatography, immunoaffinity enrichment, microbeads, and microfluidic chips. Following enrichment, the isolated EVs were detected by electron microscopy, nanoparticle tracking analysis (NTA), enzyme-linked immune sorbent assay (ELISA), fluorescence-activated cell sorting (FACS), and western blotting. (d) TEPs in blood from the plasma of patients with cancer were enriched by centrifugation. Following enrichment, platelet isolation was confirmed by electron microscopy, standard NGS, RT-PCR, and FACS.

Enriching and detecting highly pure CTCs pose a significant challenge because of their extreme rarity in peripheral blood [25]. CTCs are present in the bloodstream at extremely low concentrations, typically falling within the range of 1–10 cells per 10 ml in the majority of cancer patients. Thus, efficient enrichment of CTCs can be achieved using approaches that exploit differences between tumor cells and blood cells, including the differential expression of cell surface proteins or distinct physical properties of the cells. The combination of high-throughput and automated CTC isolation technologies with validated downstream detection assays is necessary for the routine use of CTC-based diagnostics in the clinical management of patients with cancer. Many technologies have been developed and validated for CTC enrichment, detection, and molecular characterization based on biological (e.g., expression of surface proteins/receptors) or physical (e.g., size, deformability, density, and electric charge) properties that distinguish CTCs from other cells in the blood [38,39] (Figure 2).

The enrichment of CTCs based on biological features commonly employs immunoaffinity approaches involving either the trapping of CTCs or the removal of background blood cells through positive and negative selection, respectively. Over the past 20 years, keratins, constituents of the cytoskeleton of epithelial cells, have been established as detection markers for CTCs in patients with various types of carcinomas. Many CTC assays adopt principles comparable to the Food and Drug Administration (FDA)-cleared CellSearch system, which has been the ‘gold standard’ for many years. In the CellSearch system, after epithelial cell adhesion molecule (EpCAM)-based immunomagnetic enrichment of CTCs, cells are fluorescently stained for epithelial keratins (CK8, 18 & 19) as markers for CTCs, the common leukocyte antigen CD45 as an exclusion marker, and a nuclear dye (4’,6-diamidino-2-phenylindole, DAPI) to assess cellular integrity. Suspicious events are listed in a photo gallery using automated digital microscopy [38,40,41]. Another immunomagnetic-based enrichment assay for CTCs in blood samples is the AdnaTest, which, in addition to the EpCAM-labeled ferromagnetic beads used in the CellSearch® system, includes a polymerase chain reaction (PCR) step to detect tumor-specific messenger RNA (mRNA) transcripts [42]. The AdnaTest can detect tumor-specific variants of transcripts in CTCs, such as the EpCAM, mucin 1 (MUC-1), human epidermal growth factor receptor 2 (HER-2), and androgen receptor splice variant-7 (ARV7) transcripts [43]. In addition to using antibody-labeled beads for the positive selection of CTCs, alternative methods, such as affinity-based microfluidic devices, have been used to select CTCs in various cancer types. Devices like the ‘CTC-Chip’ are designed with thousands of small antibody-labeled microposts to capture CTCs bearing specific tumor antigens from blood samples. Innovative designs of ‘CTC-Chips’ have yielded improved outcomes by prolonging the interaction time between antibody-labeled microposts and CTCs, leading to enhanced cellular entrapment [44,45]. Functional assays such as the EPISPOT (EPithelialImmunoSPOT) assay, which can detect individual circulating tumor cells, have shown success in a wide range of cancer types, including breast cancer, colon cancer, prostate cancer, and melanomas [46,47]. This approach examines the occurrence of CTCs through the evaluation of specific protein secretion, shedding, or release during short-term culture lasting 24–48 h. Metastasis-Initiating-Cells (MIC) is another functional assay to analyze the invasive properties of CTCs obtained from blood into the surrounding matrix in vivo, aiding in their characterization and providing insights into tumor staging and subtypes [48]. Counterstain markers targeting non-CTC cells, such as white blood cells and red blood cells, have also been used to enrich CTCs from blood samples, and technologies such as the EasySep depletion kit (StemCell Technologies) have been developed for this purpose. Additional approaches, such as the RosetteSep method (StemCell Technologies), incorporate an extra step involving density gradient centrifugation to enhance CTC enrichment. Negative selection methods have limitations, as there is a potential for crossover of other blood components, such as CD45-negative endothelial cells, and there is an increased risk of losing CTCs during bulk white blood cell pulldowns.

Enrichment techniques for CTCs that rely on biophysical characteristics, commonly referred to as ‘label-free’ methods, utilize the unique properties of CTCs, including their specific density, size, deformability, and electric charge, to isolate CTCs from the blood. Various techniques have been explored to identify and isolate CTCs, such as separation based on differences in physical properties compared with white blood cells. For instance, the CellSearch® PC1 system is a semi-automated microfluidic device that operates independently of the epitopes. It captures and subsequently harvests rare cells, including CTCs, from whole blood, based on their size and deformability. In addition, the Parsortix system (Angle, UK), was FDA-cleared in May 2022 for CTC enrichment in metastatic breast cancer [49]. Similarly, size-based enrichment microfluidic devices, such as 2D membrane microfilters (e.g., CellSearch®, Parsortix®, and ISET®) and 3D membrane microfilters (e.g., FaCTChecker and Resettable Cell Trap), are used to isolate single CTCs or CTC clusters based on their size and/or deformability (e.g., Cluster-Chip) [50]. However, although these methods are based on size exclusion, limitations exist when CTCs are similar in size to white blood cells. Thus, selective size amplification techniques using microbeads labeled with anti-EpCAM antibodies have been developed to increase cell recovery and purity. Differences in deformability between CTCs and normal blood cells have been exploited to isolate CTCs using microfluidic channels. Recent developments in microfluidic-based devices, such as Celsee systems, have shown higher sensitivity in detecting CTCs compared to surface marker-based systems, although challenges persist in detecting small CTCs [51]. Additionally, technologies such as the ApoStreamTM device, which analyzes surface charge and polarizability differences, have demonstrated enhanced detection and recovery of CTCs in certain cancers [52]. Post-enrichment technologies, including the ScreenCell® System, successfully isolate and recover single CTCs, enabling advanced mutational analysis through next-generation sequencing (NGS) technologies such as the Ion Torrent CellSieve™ system [53].

### Circulating cell-free tumor DNA

CtDNA is DNA that is actively secreted and/or originates from apoptotic or necrotic cancer cells and released directly into the bloodstream (or another biological fluid) [38,54,55]. It constitutes only a limited fraction of the overall circulating free DNA (cfDNA) and is the predominant source of cfDNA that arises from normal cells under physiological conditions. The majority of cfDNA fragments typically range from 143 to 180 bp in length, corresponding to the DNA length within a nucleosome [56]. These fragments exhibit a half-life in the bloodstream of 16 minutes to 2.5 hours [57]. cfDNA can be detected in body effluents, including blood, urine, cerebrospinal fluid, pleural fluid, and stool [58,59]. For studies involving blood-based analysis, plasma is generally described as a more suitable source for ctDNA analysis, with a lower background of cfDNA from non-tumor cells. ctDNA may also be derived from disrupted CTCs in the bloodstream, but its quantitative contribution to total ctDNA is unclear and likely depends on cancer stage and treatment. In 1948, Mandel and Metais were the pioneers in describing nucleotide acids in blood [60], ctDNA was first identified by Stroun et al. in 1989 [61]. Sequencing of ctDNA enables the identification of tumor mutations, distinguishing it from cfDNA. Research has shown that alterations in tumor levels extend beyond mere quantity. The sequences of ctDNA samples from the plasma of tumor-afflicted patients indicate mutations in oncogenes such as KRAS (*V-Ki-ras2 Kirsten rat sarcoma viral oncogene homolog*) [62]. Furthermore, ctDNA levels in the plasma fluctuate depending on factors such as tumor burden, tumor stage, and response to therapy [63]. In precision medicine, the clinical application of ctDNA extends beyond quantification to include the analysis of variants present in the plasma. Thus, ctDNA is a suitable biosource for analyzing cancer genome mutations for the diagnosis, prognosis, and prediction of therapeutic responses. Furthermore, as ctDNA can be released from all metastatic sites, it may represent not only the genomic landscape of the primary tumor, but also intratumor clonal heterogeneity [56].

#### Enrichment and detection technologies of ctDNA from liquid biopsies

For ctDNA processing/analysis, several factors can lead to variations in the results that compromise ctDNA clinical validation. These factors include the selection of blood collection tubes, interval between sample collection and blood processing (partially mitigated by choosing suitable tubes for collection), and decisions regarding DNA extraction methods and sample storage. Plasma samples should be processed within 6 h of collection to avoid leukocyte degradation, which might increase the amount of non-tumor cfDNA [64–67]. Several methods for extracting cfDNA exist, including centrifugation, immunomagnetic beads, silica column-based enrichment, polymer-mediated enrichment, phenol-chloroform-based extraction, and vacuum generation [68] (Figure 2). However, these approaches require additional standardization and harmonization. The selection of a method depends on the desired DNA purity and level of automation required. Given that current DNA extraction methods do not distinguish between ctDNA and cfDNA, it is imperative to follow DNA extraction for the detection of genomic variations in ctDNA. There are two primary approaches to analyzing ctDNA: targeted methods, which concentrate on specific gene rearrangements or mutations within genomic regions associated with a particular tumor type, and untargeted approaches, which comprehensively analyze and monitor the tumor genome, revealing information on nucleotide alterations, copy number aberrations, chromosomal changes, etc., without relying on prior molecular alteration data.

Advancements in technology, such as droplet-based digital PCR (ddPCR) and massive parallel sequencing (MPS) (next-generation sequencing, digital polymerase chain reaction, real-time PCR, mass spectrometry, and hypermethylation analysis), now offer an unprecedented level of sensitivity and accuracy in identifying cancer-specific genetic and epigenetic changes within biological samples, including body effluents. The exceptional precision provided by these technologies plays a pivotal role in the recent significant progress in the utilization of ctDNA in the management of cancer patients [38,54]. The selection of a specific technology is contingent upon factors such as the number of genes to be analyzed, quantity of ctDNA in a sample, nature of the genetic or genomic alteration, and cost considerations.

ddPCR involves the partitioning of sample DNA, comprising both target and background DNA, into numerous independent droplets or partitions [62]. Subsequently, the target sequence underwent endpoint PCR amplification within each droplet, and the relative proportions of positive and negative droplets were quantified by counting (using fluorescent probes). This method allows for relative quantification of target samples. In 2003, Vogelstein et al. described a new version of the technology ‘BEAMing’ (beads, emulsion, amplification, and magnetics) that uses aqueous microdroplets and beads for the detection and enumeration of genetic variants [69]. BEAMing has been successfully used in the context of liquid biopsy; however, it requires a complex experimental procedure that has limited its broad use in clinics.

ddPCR experiments offer heightened sensitivity, simpler setup compared to MPS-based methods, and faster processing without the need for intricate informatics support. However, ddPCR requires prior knowledge of the genetic or epigenetic changes. Typically, single tumor-specific mutations or a limited panel derived from prior genomic analyses are used for ddPCR. Despite nanoliter-sized compartment limitations restricting multiplexing to 3–4-plexes, alternative strategies enable the screening of mutation pools, including RAS (*Rat sarcoma virus*)/RAF (*Rapidly Accelerated Fibrosarcoma*) mutations and *epidermal growth factor receptor* (EGFR) exon 19 deletions [70,71]. By contrast, MPS provides extensive multiplexing capabilities that are theoretically capable of identifying novel genetic or epigenetic modifications. Nevertheless, MPS is time consuming and requires robust informatics support. Whole-genome sequencing (WGS) or whole-exome sequencing (WES) strategies, which are applicable for copy-number aberrations, point mutations, and other genetic anomalies, often yield low sequencing coverage and diminishing sensitivity in ctDNA analysis. Targeted MPS using cancer-specific gene panels, initially limited to approximately 2% analytical sensitivity, has been enhanced using highly sensitive and optimized procedures [72]. Innovations such as the Safe-sequencing system (Safe-SeqS), tagged-amplicon deep sequencing (TAm-Seq), and error reduction strategies such as base position error rate (BPER) analysis and cancer personalized profiling by deep sequencing (CAPP-Seq) effectively decrease detection thresholds to below 0.01% [73].

### Circulating cell-free RNA

CfRNA refers to RNA molecules that are present in bodily fluids, such as blood, and are not enclosed within cell complexes. cfRNAs are released from various cell types, including cancerous and non-cancerous cells. The analysis of cfRNA goes beyond evaluating the specific abundance of particular genes; it takes into account additional factors, such as pathogenic alternative splicing or RNA editing, which are detectable exclusively in the transcriptome, not in the genome [74,75]. Owing to these considerations, there has been an increasing focus on cfRNA over ctDNA in recent years. CfRNAs are encapsulated within extracellular membrane vesicles or form ribonucleoprotein complexes that protect them from nuclease activity [76]. Fragments of cfRNA can be obtained in multiple forms from different bodily fluids, such as serum, plasma, urine, bile, and cerebrospinal fluid [77]. Additionally, cfRNAs demonstrate resilience against RNase degradation, thereby maintaining their stability in bodily fluids [78]. This stability makes cfRNA a promising candidate for liquid biopsy-based diagnostic approaches [79]. CfRNAs encompass diverse RNA types, including microRNAs (miRNAs), transfer RNA (tRNA), piwi-interacting RNA (piRNAs), long non-coding RNA (lncRNAs), small nuclear RNA (snRNAs), small nucleolar RNA (snoRNAs), and Y RNA, which originate from various cells and tissues [80]. Extensive research within the field of cfRNA has focused on miRNAs as potential disease biomarkers in circulation, attributed to their enhanced stability in the blood [81]. Nevertheless, there is growing interest in exploring long RNAs (>200 nt), including mRNAs and lncRNAs [82]. Functioning as signaling molecules, they engage in cellular communication and potentially play a regulatory role, impacting the tumor microenvironment and influencing tumor progression and invasion [83].

#### Enrichment and detection technologies of cfRNA from liquid biopsies

The cfRNA obtained from a blood sample provides an approximation of an individual’s transcriptome at a specific moment. Consequently, the processing and analysis of cfRNA involves various factors that can introduce variations in results, including the choice of blood collection tubes, timing between sample collection and blood processing (partially alleviated by selecting appropriate blood collection tubes), and decisions regarding RNA isolation methods and sample storage [75]. The predominant method for RNA extraction from biofluids involves the use of RNA extraction kits tailored specifically for plasma and/or serum. An important concern during RNA isolation from plasma is the risk of DNA contamination, as most cfRNA isolation kits recover a fraction of cell-free DNA present in the biofluid [84,85]. Nonetheless, various techniques can be applied to alleviate these biases, including maintaining uniformity in the selection of extraction kits, assessing RNA extraction efficiency using spike-ins, examining samples with suboptimal RNA quality, and implementing measures to eliminate DNA contamination [75,86].

A consensus is lacking on the optimal method for quantifying RNA derived from blood, given that both the quantity and quality of input RNA significantly influence downstream processes. To date, the most frequently employed methods for assessing the quality and quantity of cfRNA for samples with low RNAs concentrations rely on spectrophotometry, such as the Qubit [87] and Agilent Bioanalyzer systems [88]. To quantify cfRNA, three primary methods were employed for the assessment of RNA transcripts: i) real-time quantitative reverse transcription (qRT-PCR), ii) microarray platforms, and iii) NGS (Figure 2).

### Tumor-associated extracellular vesicles

EVs are defined as particles naturally released from cells, enclosed by a lipid bilayer, and are not capable of replication [89]. EVs term utility increase due to the current limitations to precisely isolate only one vesicle type with high purity with current isolation methods [90,91]. The biogenesis of EVs is a complex and regulated process that varies among subtypes, primarily exosomes, microvesicles, and apoptotic bodies. Exosomes originate from the endosomal pathway, where multivesicular bodies (MVBs) release intraluminal vesicles as exosomes upon fusion with the plasma membrane. Microvesicles, also known as ectosomes, are formed by the direct outward budding of the plasma membrane. Apoptotic bodies, however, are larger vesicles released during programmed cell death (apoptosis). Depending on their origin, their biological functions differ. For instance, exosomes, identified as small extracellular vesicles with diameters ranging from 35 to 150 µm and originating from endosomes, play a role in establishing the pre-metastatic niche and influencing the organotropism of cancer cells [92,93]. Depending on their size and origin, they exhibit distinct physiological and pathological functions. These vesicles contain bioactive molecules such as proteins, lipids, and nucleic acids (DNA and RNA), which mirror the physiological state of the originating cells [91]. This composition makes them potential candidates for predicting treatment outcomes and prognosis, as well as delivering therapeutic agents to diseased cells through specific cell – cell interactions [94,95]. For example, cancer cells release EVs that promote tumor progression by facilitating angiogenesis, immune evasion, and metastasis [96,97]. Given their involvement in diverse cellular functions, including immune response modulation, cell signaling, and disease progression, there is a growing interest in understanding the biology of EVs, as well as the demand for the development of advanced technologies for the enrichment and detection of EVs. Understanding EV biogenesis, cargo composition, and their roles in intercellular communication has far-reaching implications for both basic research and clinical applications.

#### Enrichment and detection technologies of extracellular vesicles

As the interest in EVs continues to grow, there is a demand for accurate and sensitive enrichment and detection technologies (Figure 2).

Ultracentrifugation, particularly density‐gradient ultracentrifugation, is one of the most prevalent methods for high-purity EV isolation [98]. However, this method cannot be easily used in laboratory medicine, as it is often time consuming and has a low yield [99,100]. Size-based methods for EV isolation include ultrafiltration, employing membrane filters with predefined molecular weight cutoffs that risk vesicle breakage or deformation due to the applied force, impacting downstream analysis [101]. Size-exclusion chromatography can be used efficiently for EVs isolation. It removes high-abundance proteins and often requires multiple fractionation operations using a diversified column. This method employs a polymer to create a porous stationary phase in a column, enabling the differential elution of particles of varying sizes by gravity flow or liquid chromatography systems [102]. In flow field-flow fractionation, after the sample is introduced into the chamber, it is forced down the chamber’s length by a parabolic flow. Simultaneously, a crossflow was used to separate the particles in the sample. Larger particles were pushed closer to the walls of the chamber, where the parabolic flow was slower. Thus, the larger particles elute after the smaller particles, which remain at the center of the parabolic flow, and elute earlier [103].

Moreover, immunoaffinity capture-based techniques rely on the use of an antibody to capture EVs based on the expression of specific antigens, which can be attached to plates, magnetic beads, and microfluidic devices [104]. High-throughput droplet digital Enzyme-Linked Immunosorbent Assay (ELISA) with high sensitivity parallelizes droplet generation, processing, and analysis to achieve a throughput (~20 million droplets/min) more than 100 times greater than typical microfluidics [105]. Several commercial EVs isolation kits have been developed for this purpose. These kits include pre- and post-isolation steps for EV purification, eliminating non-EV contaminants such as lipoproteins and polymeric materials [106]. Flow cytometry is another high-throughput technique that allows the simultaneous analysis of multiple physical and chemical properties of individual EVs, enabling the characterization of surface markers, size, and complexity of EVs. This makes it a valuable tool for clinical applications and biomarker discovery [107]. To characterize and quantify isolated EVs, electron microscopy, nanoparticle tracking analysis (NTA), western blotting, proteomics, and molecular methods such as RT-PCR and NGS are used.

Despite rapid advances in EV isolation and enrichment, these technologies still face challenges for various clinical applications. Issues such as sample pre-treatment, isolation efficiency, standardization, EV heterogeneity, and most importantly, the yield of EVs cargo pose significant challenges to researchers in this field. The choice of detection technology for extracellular vesicles depends on the specific research objectives, nature of the biological sample, and desired level of detail.

### Tumor-educated platelets

Platelets are central players in hemostasis, thrombosis, immunity, inflammation, and metastasis [108]. The crosstalk between platelets and cancer cells has a myriad of pro-cancerous effects, including the promotion of tumor growth, EMT induction, cancer cell survival, adhesion, arrest, setting up of the pre-metastatic niche, and metastasis.

Platelet formation occurs mainly in the bone marrow, lungs, and even the bloodstream [109–112] from their progenitors, megakaryocytes [113]. Even though platelets lack a nucleus, their transcriptome, which is inherited from megakaryocytes, is translated into proteins [114–116] which are distributed in different platelet granules. In the presence of diverse stimuli, platelet granules are retained within the platelets, and their activation prompts exocytosis of the granular contents. A wide variety of RNA families, such as unspliced pre-RNA, mRNA, miR, ribosomal RNA, as well as a functional spliceosome for pre-RNA processing, have been characterized in platelets using advanced sequencing technologies [117–120]. During their lifespan, platelet directly interact with cancer cells via receptors, and indirectly through signaling molecules [34,121–124]. This interaction leads to platelet activation, resulting in the release of different molecules [125,126] which can provide a pro-tumor metastatic niche [127] and may induce EMT [128,129]. Direct platelet – cancer cell interaction and contact-dependent signaling by platelet-derived transforming growth factor beta (TGFβ) can activate the NF-κB pathways, resulting in the acquisition of mesenchymal features by cancer cells and increased metastasis potential [130]. Jiang et al. demonstrated the presence of platelet-coated CTCs in metastatic cancer patients [131]. Introducing platelet-educated tumor (PETs) concepts showed that platelets can impact cancer cells by highly efficient transfer of lipids, proteins, and RNA through different mechanisms such as direct contact, internalization, or via EVs [132]. Recently, using a permanent CTC line, we demonstrated that incubation with a CTC-conditioned medium induced platelet aggregation and activation. This supports the hypothesis that the interaction between CTCs and platelets plays a role in preserving CTC integrity during circulation in the bloodstream [128]. The clinical aspects of platelets in liquid biopsies were highlighted by Best et al. [133]. In this study, platelet RNA profiles from healthy individuals and patients with cancer were subjected to machine learning, resulting in the development of a classification algorithm for pan-cancer diagnosis, determination of tumor location, and identification of oncogenic alterations driving the disease [133,134]. Most recently, in a larger cohort, the presence of cancer in two-thirds of samples with 99% specificity highlighted the potential properties of TEP-derived RNA panels to supplement current approaches for blood-based cancer screening [135]. In addition to transcriptomics, platelet proteomics showed a significant difference between early stage patients and healthy samples [136].

#### Enrichment and detection technologies of TEPs

Platelet isolation is easy; however, it is very important to prevent their activation during handling because this affects their molecular and morphological features (Figure 2). Therefore, strong mechanical or biochemical forces should be avoided to obtain intact platelets [137]. However, it is essential to optimize a quick and effective technique to isolate platelets from a small volume of blood. Blood should be carefully drawn using a 1.2 mm intravenous cannula to reduce platelet activation during blood sampling [138].

As different medications affect platelet function, it is critical to accurately document every treatment a patient receives. TEPs can be isolated up to 48 h after blood sampling, allowing the production of high-quality RNA for molecular tests [133,134]. NGS technologies have revolutionized the genomic analysis of TEPs, allowing for comprehensive profiling of RNA, including coding and non-coding transcripts. Whole-transcriptome sequencing of TEPs enables the identification of tumor-derived signatures, offering insights into the molecular landscape of the originating tumor [133,134]. Assessing the responsiveness of platelets to various stimuli is essential, and functional assays, such as the aggregation test and flow cytometry, play a crucial role in revealing the functional implications of molecular changes [139–141]. Through in-depth and quantitative analysis of platelet protein composition, a comparative evaluation of the structural and functional pathways was performed. It also helps to better understand the physiological and pathophysiological processes related to homeostasis and thrombosis [142]. Understanding the molecular mechanisms of platelets will be aided by the integration of transdisciplinary approaches such as transcript omics, metabolic analysis, and bioinformatics with recent advances in proteomic technology. Thus, a thorough examination of the platelet proteome in various environmental settings may help clarify the intricate mechanisms pertaining to platelet function in relation to diseases or platelet hyperreactivity and provide novel targets for antiplatelet medication [143].

## Clinical applications of liquid biopsy

Non-invasive blood samples can be obtained repeatedly for *(i)* predicting relapse in M0 patients or metastatic progression in patients with advanced cancer; *(ii)* staging and stratifying patients for therapy; *(iii)* monitoring the efficacy of therapies and discriminating early responders from nonresponders, tracking tumor evolution, and identifying resistance mechanisms), *(iv)* early detection of minimal residual disease.

### Prognostic indicator

Assessing the risk of adverse outcomes in patients during or after treatment is essential for determining the appropriateness of more aggressive therapy and the frequency of monitoring. Prognostic evaluation was conducted for both ctDNA/cfDNA and CTCs.

The clinical significance of CTCs as biomarkers for treatment response and prognosis has been extensively investigated. In particular, in patients with advanced breast or prostate cancer, the sequential enumeration of CTCs in the context of systemic therapies provides early and reliable prognostic information. De Bono et al. [144] demonstrated that CTC enumeration is a superior indicator of treatment response than a 50% reduction in serum prostate-specific antigen (PSA) level. Moreover, using data from five randomized trials including 6,081 metastatic castration-resistant prostate cancer (mCRPC) patients, Heller et al. found that the absence of CTCs and conversion from ≥ 5 CTCs at baseline to ≤ 4 CTCs after 2 weeks had the strongest discriminating power for predicting overall survival (OS) [145]. Consequently, CTCs have been proposed as surrogate endpoints in clinical trials that assess OS in patients with mCRPC [146]. For breast cancer, sequential CTC enumeration has been shown in a large multicenter prognostic study to be superior to conventional serum protein markers (CA-15-3, CEA) for the early detection of therapy failure [147]. However, among metastatic breast cancer (MBC) patients with persistent CTCs increase after 21 days of therapy, the CTC-driven switch to an alternative cytotoxic therapy did not prolong OS in the interventional trial SWOG 0500 (NCT00382018) [148]. However, in the recent STIC-CTC trial (NCT01710605), the reverse approach consisting of identifying patients who may not need aggressive treatment based on their low CTC count showed that the CTC count is a reliable biomarker for choosing between chemotherapy and endocrine therapy as the first-line treatment for hormone receptor – positive (HR+) HER2− metastatic breast cancer (MBC) [149]. The clinical effectiveness of CTCs has been established for the first time in this field.

The prognostic potential of CTC enumeration in metastatic non-small cell lung cancer (NSCLC) was assessed through a pooled analysis of patients from nine European NSCLC CTC centers. These results confirmed that CTC counts were associated with reduced progression-free survival (PFS) and OS [150]. Moreover, in patients with advanced-stage NSCLC after one cycle of chemotherapy, CTC-based surveillance revealed that the prognosis was worse in patients with >5 CTCs than in those with < 5 CTCs. The number of CTCs could be modulated by therapeutic intervention, and lower CTC counts were correlated with better clinical outcomes [151].

The quantification of ctDNA sheds could offer robust prognostic information for patients with advanced cancer across multiple tumor types.

To ascertain whether the percentage of ctDNA corresponded with clinical outcomes, Schwaederle et al. [152] assessed ctDNA levels in a cohort of 168 patients with diverse cancers. Patients’ OS and PFS were considerably poorer when they had at least one gene alteration with >5% ctDNA. Different pan-cancer analyses confirmed shorter clinical outcomes in the presence of ctDNA. Vu et al. found an independent negative correlation between higher total number of ctDNA alterations and poorer OS [153]. Comparing ctDNA and tissue DNA, patients with similar TP53 mutations in tissue and ctDNA had significantly shorter survival than patients with different mutations or no mutations [154].

Ikeda et al. investigated mesenchymal – epithelial transition (MET) alterations in a range of tumor types using ctDNA and discovered that MET alterations were associated with a considerably shorter time to metastasis or recurrence, as well as lower OS [155]. A higher total percentage of ctDNA was also reported to be a predictive factor for worse survival in a study on pancreatic cancer patients by Patel et al. [156].

In their evaluation of preoperative ctDNA in peritoneal carcinomatosis patients, Baumgartner et al. discovered that, regardless of histologic grade, individuals with high levels of ctDNA had a shorter PFS following surgery [157]. When ctDNA was detected in patients with triple-negative breast cancer who were undergoing or completed neoadjuvant chemotherapy, the disease-free survival (DFS) was considerably reduced. The presence of ctDNA predicts OS and DFS before surgery [158]. Postoperative ctDNA presence in a cohort of patients with locally advanced rectal cancer indicated disease recurrence regardless of the administration of adjuvant chemotherapy and evidence of pathological complete response [159]. Anandappa et al.’s study on post-operative stage II-III patients not undergoing adjuvant chemotherapy found a significant association between positive ctDNA and higher relapse rates [160].

#### Stratification of patients for therapy

Establishing actionable alterations in metastatic solid tumors is essential for implementing a precision medicine treatment approach. Traditionally obtained through molecular profiling of tumor tissue biopsies, ctDNA/cfDNA and CTCs are emerging as valuable alternatives in this context.

CTCs play a role in the identification of patients undergoing hormone therapy [161]. A recent study in patients with metastatic hormone-sensitive prostate cancer (mHSPC) from the SWOG S1216 trial showed that patients without CTCs were significantly more likely to attain complete biochemical response and had a significantly lower risk of disease progression and death after adjusting for clinical covariates than patients with ≥5 CTCs. This suggests that the baseline CTC count is a valuable prognostic marker in patients with mHSPC at therapy initiation to discriminate between patients with favorable and unfavorable responses and OS [162].

Magbanua et al. developed a novel latent mixture model to stratify patients with similar CTC trajectory patterns during chemotherapy. This approach revealed distinct prognostic subgroups among patients with poor prognosis, suggesting the potential benefits of more targeted treatments. This prognostic classification approach can be used to refine CTC-based risk stratification strategies and guide future prospective clinical trials in patients with MBC [163].

A continuously growing body of evidence indicates that CTC HER-2 or estrogen receptor (ER) status can be different from that of the corresponding primary tumor in breast cancer patients. Moreover, the HER-2 or ER status of CTCs can change over time, particularly during disease recurrence or progression [164–167]. Clinical trials are now underway to investigate whether patients with HER2− primary tumors but HER2+ CTCs will benefit from HER2-targeting therapies, such as lapatinib (DETECT-III study, NCT01619111) or trastuzumab (CTC-TREAT study, NCT01548677). To predict resistance to endocrine therapy in patients with HR+ MBC, Paoletti et al. developed a multiparameter CTC-Endocrine Therapy Index (CTC-ETI) using the CellSearch® system [168]. The CTC-ETI integrates both the counting of CTCs and evaluation of CTC expression for four markers: ER, B-cell lymphoma-2 (BCL2), HER2, and Ki67. The clinical relevance of the CTC-ETI is being evaluated in an ongoing clinical trial (NCT01701050).

In metastatic colorectal cancer (mCRC), the chemotherapy regimen FOLFOXIRI plus bevacizumab is more effective than FOLFOX/FOLFIRI plus bevacizumab as the first-line therapy. However, it is not widely used because of concerns regarding toxicity and the lack of predictive biomarkers. Analysis of the role of CTC counts as a biomarker for patient selection showed that in patients with a baseline CTC count ≥ 3, first-line FOLFOXIRI-bevacizumab significantly improved PFS compared with FOLFOX-bevacizumab [169]. Thus, CTC counts may help select patients for intensive first-line therapy.

cfDNA analysis allows for comprehensive genomic profiling of tumors. By identifying specific genetic alterations, such as point mutations, copy number variations, and rearrangements, clinicians can gain insight into the molecular characteristics of the tumor. This information is crucial for selecting targeted therapies tailored to the genomic profile of an individual.

FDA has approved several blood-derived ctDNA-based companion diagnostic tests. These include the Cobas EGFR mutation test V2, which can identify EGFR mutations in non-small cell lung cancer, and the Therascreen PIK3CA RGQ PCR kit, which can identify PIK3CA mutations in breast cancer [170]. Additionally, the FDA has approved FoundationOne liquid biopsy and Guardant360 liquid, both of which are based on NGS of ctDNA, as companion diagnostics for various targeted therapies and for general molecular profiling. These diagnostics may affect patients’ therapeutic decisions or qualify them for clinical trials.

Numerous studies have investigated additional ctDNA testing for druggable changes. One such disease that can be difficult to treat is tumors with unknown primary (CUP). These patients usually have poor overall results from empirical chemotherapy using regimens based on taxanes and/or platinum [171]. Kato et al. used ctDNA NGS to assess 442 patients with CUP [172]. They discovered that 80% showed ctDNA abnormalities, 66% showed ≥1 characterized alteration, and almost all (>99%) exhibited potentially targetable alterations. In a further study, the same group, examined 1931 CUP patients using cfDNA NGS panels [173]. In a subset of patients with clinically accessible data, those with higher degrees of matching between drugs and molecular alterations had significantly improved clinical benefit rates compared with those with lower degrees of matching, indicating clinical utility.

ctDNA/cfDNA can also aid in identifying possible actionable mutations in other cancer types. In a study of 62 individuals with metastatic breast cancer, Shatsky et al. discovered that 68% had at least one potentially actionable alteration [174]. In gynecologic malignancies, Charo et al. found that therapy tailored to ctDNA-identified mutations resulted in significantly better survival than unmatched therapy [175]. In a cohort of patients with colorectal cancer, Choi et al. discovered at least one actionable mutation in 76% of patients. All characterized alterations were potentially targetable with FDA-approved or experimental drugs in clinical trials [176]. Okamura et al. showed that 76% of 121 patients with biliary tract malignancies who received systemic treatment had at least one ctDNA alteration. Among these, 80 patients underwent treatment, and those who received molecularly matched therapy based on genomic profiling through ctDNA and/or tissue DNA experienced significantly extended progression-free survival and a higher disease control rate compared to individuals on unmatched regimens [177].

#### Monitoring treatment response and mechanism of resistance

CTCs and cfDNA/ctDNA can serve as dynamic biomarkers that reflect the response of tumors to therapeutic interventions. Monitoring changes during the course of treatment provides clinicians with valuable information regarding the efficacy of specific therapies. This real-time feedback enables prompt adjustments to treatment plans, optimizes patient care, and potentially avoids unnecessary side effects of ineffective treatments.

In addition to the CTC number, CTC molecular phenotypes have a strong prognostic value. For instance, programmed death-ligand 1 (PD-L1) expression in CTCs of patients with advanced NSCLC has been associated with a poor prognosis. The differential expression of PD-L1 and Ki67 in CTCs may yield additional predictive information for individuals with advanced NSCLC who have been administered pembrolizumab. Changes in the PD-L1low subpopulation during early treatment have been associated with disease control (decreased number) and resistance to immunotherapy [178].

The androgen receptor (AR) is a key target in prostate cancer, and many current therapies for metastatic castration-resistant prostate cancer (mCRPC) target AR signaling [161]. As first shown by Antonarakis et al., the androgen receptor splice variant 7 (AR-V7) is frequently present in CTCs of patients with mCRCP, and it predicts therapy resistance against Enzalutamide and Abiraterone, both agents that block the AR pathways [179]. In a more recent multicenter prospective blinded study, Armstrong et al. found that the presence of CTCs AR-V7(+) before treatment was independently associated with worse PFS and OS, as well as with a lower probability of confirmed PSA responses during treatment with abiraterone or enzalutamide [180]. These findings indicate that CTC AR-V7 status has prognostic relevance. Furthermore, in breast cancer, the presence of full-length AR-positive CTCs and CTCsAR-V7(+) is associated with therapeutic failure, suggesting that AR inhibition may not be effective in primary breast cancer [181]

Platinum resistance is one of the most recognized clinical challenges in ovarian cancer. Molecular analysis of CTCs in ovarian cancer indicates resistance to platinum-based treatments. Although immunohistochemistry of ERCC1 protein in primary tumors did not predict platinum resistance, ERCC1(+) CTCs did predict platinum resistance at the primary diagnosis of ovarian cancer [182]. A meta-analysis of eight studies, including 1,184 patients, showed that patients with ERCC1(+) CTCs had significantly shorter OS and DFS than those with ERCC1(−) CTCs [183]. Changes in cfDNA levels and genetic profiles during treatment offer real-time insights into treatment response. Monitoring the emergence of new mutations or alterations provides early warning of potential resistance to ongoing therapies.

Mutations in EGFR have been a key focus of ctDNA liquid biopsies, as these mutations dictate the efficacy of EGFR-targeted therapies in patients with NSCLC. For example, sensitivity to the EGFR tyrosine kinase inhibitors erlotinib and gefitinib is dependent on the presence of activating aberrations such as the EGFRL858R mutation or exon 19 deletions, whereas the EGFRT790M mutation is associated with resistance to these agents.

Sequential ctDNA examination was performed by Ortiz-Cuaran et al. in patients with BRAF-mutated NSCLC undergoing BRAF-directed treatments. In this study, mutant ctDNA clearance was associated with longer PFS and OS relative to that in patients with detectable levels of early mutant ctDNA. These results indicate that early ctDNA measurement may indicate treatment response, whereas ongoing monitoring of BRAF ctDNA levels could be a clinically useful marker for tumor response, similar to observations in EGFR-mutant NSCLC patients [184].

In another analysis of plasma ctDNA from patients involved in the PALOMA-3 study (*n* = 195), O’Leary et al. showed that clonal evolution frequently occurs during treatment, reflecting substantial subclonal complexity in breast cancer that has progressed after prior endocrine therapy, and new driver mutations emerged in PIK3CA and ESR1 (in particular, ESR1 Y537S) after treatment with either palbociclib plus fulvestrant or placebo plus fulvestrant [185].

Mutations in the androgen receptor (AR) gene have been detected in CTCs of patients with castration-resistant prostate cancer (CRPC) [186]. Many of these mutations were also detected in tumor specimens and were associated with resistance to androgen deprivation therapy. Similar information can be obtained from the profiling of ctDNAs from patients with prostate cancer [187].

KRAS mutations have an adverse impact on the efficacy of anti-EGFR antibody therapy in patients with CRC. Sequential ctDNA analysis during EGFR inhibition revealed that KRAS and NRAS mutations can rapidly emerge because of the selective pressure exerted by targeted therapy [188]. Interestingly, it has been proposed that the emergent population of KRAS-mutant subclones declines upon withdrawal of anti-EGFR therapy, suggesting guided ‘cyclical therapy,’ characterized by sequential withdrawal and reintroduction of EGFR inhibitors based on genetic data from ctDNA analyses [188].

#### Early detection of minimal residual disease (MRD)

After primary treatment, detection of residual disease is crucial for predicting the likelihood of disease recurrence. CTCs and ctDNA offer a means of detecting minimal residual disease, helping clinicians identify patients at a higher risk of relapse. This information guides decisions regarding the duration of therapy, the need for additional treatments, or the intensity of post-treatment surveillance, improving treatment outcomes and overall survival rates.

CTC characterization based on gene expression, DNA methylation, and DNA mutation analysis, in combination with CTC count and phenotypic analysis, can identify MRD up to years before clinically detectable metastatic disease [189]. Interestingly, the detection of prostate-specific membrane antigen (PSMA)-positive CTCs in patients with non-metastatic triple-negative breast cancer before and after neoadjuvant chemotherapy has shown clinical significance in identifying patients at a high risk for relapse [161].

In early stage NSCLC, the TRACERx study investigated CTC enumeration in pulmonary vein blood samples (collected during surgery) using the CellSearch® system. These CTCs represent subclones responsible for tumor relapse and remained an independent predictor of relapse in the multivariate analysis adjusted for tumor stage [190]. Genomic profiling of single CTCs isolated from pulmonary vein blood samples collected during surgery revealed that their mutation profile was more similar to that of metastasis detected 10 months later than the primary tumor mutation profile [190]. Additionally, the risk of nodal and distant tumor recurrence is increased [191]. Higher pre-treatment CTC counts and CTC persistence after treatment were significantly associated with an increased risk of recurrence outside the targeted treatment site [191].

In patients with non-metastatic CRC, Van Dalum et al. prospectively monitored CTC changes using the CellSearch® platform for a median of 5.1 years after the initial diagnosis [192]. They found that the presence of CTCs after surgery and before initiation of adjuvant therapy did not influence clinical outcomes. Conversely, CTC detection 2–3 years after surgery predicts unfavorable prognosis. Therefore, CTC detection may suggest CRC recurrence and the long-term persistence of MRD. Wang et al. showed that patients with early stage CRC and detectable CTCs after surgery had a significantly higher risk of recurrence, and thus reduced recurrence-free survival rates. In early stage CRC, patients with a preoperative CTC count of ≥4 exhibited a significantly higher recurrence risk than those with <4 CTCs [193]. Additionally, they noted that the recurrence rate reached 100% when the postoperative CTC count remained ≥4 for more than three consecutive time points within–2-6 month period, regardless of the patient’s clinical risk status and preoperative CTC levels [194].

In 2015, Garcia-Murillas et al. offered initial insights into the application of ctDNA measurements for monitoring MRD in patients with high-risk early stage breast cancer [195]. In this study, post-surgery blood samples from 55 patients were analyzed for the presence of somatic mutations known to be present in their primary tumors, and the detection of ctDNA was correlated with an increased risk of metastatic relapse [195]. In a secondary analysis of a phase 2 multicenter randomized clinical trial that randomized patients with early stage triple-negative breast cancer (TNBC) who had residual disease after neoadjuvant chemotherapy to receive post-neoadjuvant genomically directed therapy versus treatment of physician choice, Radovich et al. investigated whether the independent presence of ctDNA and CTCs after neoadjuvant chemotherapy in patients with early stage TNBC was associated with recurrence and clinical outcomes [196]. Among 196 female patients, ctDNA detection was significantly associated with inferior distant disease-free survival (DDFS) and disease-free survival. Furthermore, the concurrent presence of ctDNA and CTCs provides additional information, thereby augmenting sensitivity and discriminatory capacity. Patients displaying positivity for both ctDNA and CTCs exhibited significantly poorer DDFS than those who tested negative for both ctDNA and CTCs.

In stage II – III CRC, the postoperative 3-week ctDNA MRD status was a better indicator of recurrence than other established clinicopathological risk factors [197]. Furthermore, a distinct distinction in the clinical results was noted between patients whose ctDNA MRD persisted following curative surgery or adjuvant therapy and those whose ctDNA MRD was resolved by adjuvant therapy, highlighting the significance of ctDNA MRD as an indicator of chronic illness [198,199].

In early stage NSCLC, Gale et al. observed that the identification of ctDNA preceded the clinical detection of primary tumor recurrence by a median of 212.5 days [200]. Among the patients experiencing clinical recurrence of their primary tumor, ctDNA was detected post-treatment in 18 of 28 individuals (64.3%). Detection within the pivotal period of 2 weeks to 4 months following the conclusion of treatment was noted in 17% of patients, and this was linked to reduced recurrence-free survival (RFS). Furthermore, MRD-positive patients who received adjuvant therapies had improved RFS compared to those who did not receive adjuvant therapy, whereas MRD-negative patients who received adjuvant therapies had lower RFS than their counterparts without adjuvant therapy [201].

## Diagnosis, monitoring, and prognosis of liquid biopsy in cancer immunotherapy

Immuno-oncology, encompassing checkpoint inhibitors represents the latest breakthrough in the field and stands as a significant advancement in the ongoing fight against diverse cancers such as metastatic melanoma [202], kidney cancer [203], bladder cancer [204], NSCLC [205,206], small cell lung cancer [207], gastric cancer [208], breast cancer [209], liver cancer [210], and more. These advancements have been attributed to the increasing development of novel immunotherapies and the corresponding expansion of clinical trials. The growing significance of liquid biopsy in clinical practice for cancer diagnosis and prognosis is attributed to its simplicity, non-invasiveness, high specificity, and ability to overcome temporal-spatial heterogeneity [11]. Below, we discuss the application of various liquid biopsy biomarkers (CTC, ctDNA, cfRNA, EVs, and platelets) in the diagnosis, monitoring, and prognosis of cancer immunotherapy.

CTCs exhibit significant potential as immunotherapeutic biomarkers, owing to their gradual increase in abundance throughout disease progression [211]. The discovery of PD-L1 expression in CTCs has generated interest in elucidating their relevance within the framework of immune checkpoint inhibitor therapy. Moreover, the quantification and qualitative evaluation of CTCs in individuals undergoing immune checkpoint inhibitor therapy suggest that the analysis of these cells before and after treatment could provide prognostic value in predicting overall survival and treatment response [212]. In malignant melanoma, qualitative examination of CTCs revealed that pre-treatment PD-L1-positive CTCs predicts responsiveness to anti-PD-1 immune checkpoint inhibitors [213,214]. In addition, in lung cancer, the identification of PD-L1 on CTCs in NSCLC has been reported as a potential indicator of reduced overall survival [215]. Another study revealed an unfavorable prognosis for patients with PD-L1-positive CTCs, noting that the persistence of PD-L1-positive CTCs after nivolumab therapy correlates with diminished overall survival [216]. Although immune checkpoint inhibitors have not been thoroughly explored in breast carcinomas, Mazel et al. revealed the presence of PD-L1 in 68% of breast cancer CTCs [217]. In addition, Schott et al. found PD-L1-positive CTCs in both early and metastatic disease stages, reporting a case in which successful nivolumab and ipilimumab treatment led to a reduction in PD-L1-positive CTCs [218]. Various malignancies, including prostate, bladder, colon, head and neck squamous cell carcinoma, and hepatocellular carcinoma, are being scrutinized for CTCs as immunotherapeutic biomarkers [218–220]. For instance, in hepatocellular carcinoma, the presence of PD-L1^+^ CTCs is a predictor of worse overall survival but predicts a positive response to nivolumab treatment [221].

Besides CTCs, cfDNA, or more specifically ctDNA, is the most important source in LBs, and consequently, efforts have been made to implement cfDNA in the field of immuno-oncology. For instance, in a study by Cabel et al., a significant correlation between simultaneous alterations in ctDNA levels and tumor size was identified after the initial immunotherapy injection [222]. Additionally, Leprieur et al., who investigated advanced NSCLC patients receiving nivolumab treatment, revealed that low ctDNA levels were associated with long-term benefits of nivolumab [223]. Goldberg et al. observed that a reduction in cfDNA levels served as an early indicator of therapeutic success and predictor of extended survival. Interestingly, the amount of cfDNA in the bloodstream can also be used to monitor NSCLC patients undergoing immunotherapy [224]. Consequently, liquid biopsy based on ctDNA has gained popularity in selecting treatment regimens for advanced NSCLC, aiding in the identification of therapeutic drug targets and the detection of immunotherapy biomarkers, such as blood tumor mutational burden and blood microsatellite instability [225]. Simultaneously, in melanoma cancer, Ashida et al. investigated the correlation between ctDNA and anti-PD-1 immunotherapy, revealing a decline in ctDNA levels after 2–4 weeks in three responsive patients out of five, while remaining elevated in two unresponsive patients [226]. Although ctDNA serves as a unique tool for assessing immunotherapy efficacy, addressing limitations in sensitivity, quantification, and standards is crucial. Ongoing immune clinical experiments on ctDNA with small sample sizes are yet to yield conclusive results. Prospective immunologic clinical trials welcome participants with diverse malignancies, various clinical stages, and multiple gene mutations, with the expectation that ctDNA testing will soon become the standard method for evaluating immunotherapy efficacy.

In cfRNAs, miRNAs hold potential as both biomarkers and therapeutic targets in the context of immunotherapy [227]. For instance, in lung cancer, a correlation was observed between the downregulation of circulating miRNA expression and response to immunotherapy [228]. In addition, Peng et al. demonstrated that non-small cell lung cancer patients display unique plasma exosomal miRNA profiles, with hsa-miR-320d, hsa-miR-320c, and hsa-miR-320b identified as potential biomarkers for predicting immunotherapy efficacy [229]. In addition, treatment-induced downregulation of the T-cell suppressor hsa-miR-125b-5p may enhance T-cell function, contributing to a positive immunotherapy response [229]. Nevertheless, the available data on miRNAs are inadequate for drawing meaningful conclusions about their potential role in predicting responses and survival during immunotherapy, given the limited sample size in current studies and the diversity of methodologies employed.

Beyond EVs substantial role in tumor progression and interactions between tumors and the immune system, they are gaining attention for their ability to potentially predict immunotherapy response. The immunotherapeutic potential of EVs was first documented in the late 1990s by Zitvogel et al. They revealed that EVs derived from dendritic cells not only elicited specific cytotoxic activity in T lymphocytes *in vitro* but also demonstrated the ability to suppress tumor growth *in vivo* [230]. Moreover, different research groups have investigated the significance of biomarkers expressed by EVs in determining which patients will benefit from immunotherapy. For instance, according to Miguel‑Perez et al., PD-L1-positive EVs can be employed as prognostic biomarkers for patients with NSCLC receiving immunotherapy. In a retrospective and prospective independent cohort of 33 and 39 patients, an increase in EV PD-L1 was observed in nonresponders compared to responders, and was an independent biomarker associated with reduced progression-free survival and overall survival. Interestingly, in contrast, the commonly utilized biomarker, tissue PD-L1 expression, was not predictive of durable response or survival. This emphasizes the potential of EV PD-L1 to stratify patients with advanced NSCLC who are likely to experience lasting benefits from immune checkpoint inhibitors [231]. Additionally, EVs may serve as conduits for cancer cells to evade immune monitoring, resulting in immunotherapy failure. Tumor EVs are thought to promote lymphocyte activation by exposing inhibitory ligands that trigger an immunological checkpoint response [232]. To integrate EVs into clinical practice, numerous ongoing clinical trials are assessing their impact on immunotherapy in combination with other anticancer treatments [232]. For instance, the NCT03985696 clinical trial seeks to investigate the relevance of exosomes in response to immunotherapies by analyzing exosomal levels of CD20 and PD-L1 isolated from diffuse large B cell lymphoma cells. In the Prospective longitudinal cohort ALCINA 2, NCT-04025541, 60 lung cancer patients in the context of immunotherapy (baseline +3 follow-ups) were included. The goal was to assess the correlation between the response to immunotherapy (progressive versus non-progressive) and liquid biopsy analytes, CTC, and EVs expressing the PD-L1 marker. Indeed, ALICINA2 clinical trials were started to highlight our previous proof-of-concept study, which focused on the prognostic value of different liquid biopsy biomarkers (CTCs, PD-L1^+^ CTCs, sEVs, PD-L1^+^ sEVs, and ctDNA) alone, and in combination, in a cohort of patients with NSCLC, regardless of cancer treatment, subtype, and stage, to determine whether their combination provides more precise prognostic information [233].

Additionally, the utilization of EVs as biological transporters in cancer immunotherapy has recently emerged as an area of research. EVs modified through biotechnological methods can encapsulate specific cargoes, such as therapeutic molecules or diagnostic markers, and are designed to target particular cells or tissues. For instance, immune checkpoint inhibitors include antibodies targeting PD-1, PD-L1), and cytotoxic T-lymphocyte-associated protein 4 (CTLA-4) loaded in EVs and can stimulate an immune response, leading to the targeted elimination of cancer cells [234,235].

In addition to the importance of platelet count and cancer-associated thrombosis, revealing the profile of TEPs and their factors can foster immunotherapy development [236]. For instance, it has been reported that a higher rate of venous thromboembolism was observed in NSCLC patients treated with chemotherapy than in those receiving immune checkpoint inhibitors [237]. Moreover, the development of thrombocytopenia and low platelet count is associated with improved overall survival in metastatic patients treated with immune checkpoint inhibitors compared to patients without thrombocytopenia [238]. Cancer cells induce the release of platelet granules and extracellular vesicles to promote their survival in the bloodstream. In turn, the platelet profile is reprogrammed toward attenuate antitumor immunity via inhibition of T lymphocytes and NK cells and macrophage polarization toward M2 tumor-associated macrophages (TAMs) [236]. TGF-β, a crucial cytokine for fostering immunosuppression in the tumor microenvironment derived from platelets, reduces natural killer group 2D (NKG2D) receptor expression and inhibits their antitumor reactivity [239].

A recent study indicated that utilizing blood platelets engineered to deliver an immunotherapy drug could efficiently eradicate cancer cells overlooked during surgery and hinder their ability to develop new tumors [240]. In experiments involving *in vivo* model with surgically diminished melanoma and breast cancer tumors, mice treated with engineered platelets exhibited diminished tumor regrowth and metastasis, leading to prolonged survival compared to mice treated with normal platelets or the checkpoint inhibitor alone [240]. Their findings show the potential of platelets as targeted drug carriers because these small cell fragments accumulate in wounds; therefore, there is a potential for interaction with circulating metastatic cancer cells in the bloodstream. Moreover, when platelets are activated at the wound site, they release chemicals that enhance the local immune response, contributing to wound repair. Another recent study highlighted the responsiveness of platelets and platelet derivatives to thrombosis, to enhance the local accumulation of immune checkpoint inhibitors and chemodrugs [241]. Moreover, Rachidi et al. reported that targeting platelets enhanced the effectiveness of adoptive T-cell therapy in various cancers in mice. Their findings indicated that the presence of TGFβ from platelets significantly diminishes T cell (CD4^+^ and CD8^+^ T cells) function, primarily through the expression of the TGFβ-docking receptor glycoprotein A repetitions predominant (GARP). These results suggest that integrating platelet inhibitors with immunotherapy could serve as a supplementary strategy for cancer treatment [242].

It has been shown that platelet PD-L1 reflects collective intratumoral PD-L1 expression and can predict immunotherapy response in NSCLC. Indeed, blood platelets interact with lung cancer cells, and the PD-L1 protein is transferred from tumor cells to platelets in a fibronectin 1, integrin α5β1, and GPIbα-dependent manner. Platelets from NSCLC patients were found to express PD-L1, and platelet PD-L1 possesses the ability to inhibit CD4 and CD8 T cells, confirming their important roles in tumor immune evasion and overcoming the limitations of histological quantification of heterogeneous intratumoral PD-L1 expression [243].

## Discussion

Real-time liquid biopsy plays a pivotal role in advancing cancer management [244–246]. CTCs, ctDNA, cfRNA, EVs, and TEPs offer complementary insights, suggesting their potential integration into clinical practice in the near future [247]. However, using it in clinical routine faces several challenges. One of the key limitations of using these biomarkers is their low sensitivity. This is due to low quantities of CTCs, ctDNA, and EVs in blood and other body fluids. Platelets, even if abundant can impede with EV isolation due to their similar size and density.

Furthermore, liquid biopsies lack quality control methods and standardized techniques. All liquid biopsy biomarkers are subject to degradation during sample collection, handling, processing, and storage, which can result in loss or change of the contents. The stability of these components is essential for accurate analysis. Other analytes, such as leukocytes, cell-free DNA, lipoproteins, and protein aggregates, can also contaminate the liquid biopsy biomarker analysis. Ensuring the specificity of isolation procedures is critical for reducing contamination and increasing the reliability of liquid biopsy tests. Hence, obtaining consensus on best established methods and applying quality control are vital for ensuring the reproducibility and reliability of outcomes.

Moreover, CTCs, EVs, and platelets exhibit heterogeneity in their cargo, including proteins, nucleic acids, and lipids. This heterogeneity complicates the interpretation of results and necessitates the development of techniques capable of capturing this diversity. The development of cost-effective methods and procedures is also important for the widespread adoption of liquid biopsy.

To tackle these obstacles, multidisciplinary cooperation and continuous innovation in isolation strategies, analytical approaches, and quality control norms are needed. The potential of liquid biopsy biomarkers as useful resources for cancer diagnosis, prognosis, and treatment monitoring will be increased if these challenges are overcome. Furthermore, combining data from many biomarkers may result in the creation of an index or algorithm that improves tumor profiling accuracy. It is important to emphasize that liquid biopsy should not be perceived as a substitute for histopathological diagnosis through tissue biopsies. Instead, they should be viewed as a supplementary tool for diagnosis and characterization, contributing to an innovative approach to tumor management. For instance, CTCs may prove instrumental in identifying new therapeutic targets to impede metastatic initiator cells, whereas EVs could serve as delivery vehicles for targeted therapies in specific tissues or tumors. The presence and biological functions of these biosources extend beyond cancer, opening avenues for diverse applications that may not necessarily be directly linked to diseases or human well-being. The concept of ‘liquid biopsy’ is anticipated to become indispensable for oncologists and physicians, exemplifying a transformative shift in cancer management in the future.

## Data Availability

Data sharing is not applicable to this article as no new data were created or analyzed in this study.
